# Impact of COVID-19 on selected essential public health services – lessons learned from a retrospective record review in the Free State, South Africa

**DOI:** 10.1186/s12913-023-10166-7

**Published:** 2023-11-11

**Authors:** Christo Heunis, Perpetual Chikobvu, Michel Muteba, Gladys Kigozi-Male, Michelle Engelbrecht, Providence Mushori

**Affiliations:** 1 Centre for Health Systems Research & Development, University of the Free State, Bloemfontein, South Africa; 2 Free State Department of Health, Bloemfontein, South Africa; 3 Department of Community Health, University of the Free State, Bloemfontein, South Africa; 4 World Health Organization, Bloemfontein, South Africa

**Keywords:** COVID-19, Impact, Essential public health services, Retrospective record review, Lessons

## Abstract

**Background:**

In an attempt to discern lessons to improve future pandemic responses, this study measured the effects of the COVID-19 pandemic on essential public health services (EPHSs) related to primary health care (PHC) and outpatient department (OPD) utilisation, antiretroviral treatment (ART) commencement, drug-susceptible tuberculosis (DS-TB) confirmation and treatment commencement, and Bacillus Calmette-Guérin (BCG) coverage, in the Free State province of South Africa during January 2019 to March 2021.

**Methods:**

A pre-post study design comparing EPHS performance between 2019 and 2020/21 was employed. Routinely collected data were analysed. An interrupted time series analysis was used to measure changes in service use and outcomes from January 2019 to March 2021. Median changes were compared using Wilcoxon rank-sum tests. A 5% statistical significance level was considered.

**Results:**

Over the study period, the median values for the annual number of PHC visits was 1.80, 55.30% for non-referred OPD visits, 69.40% for ART commencement, 95.10% and 18.70% for DS-TB confirmation and treatment commencement respectively, and 93.70% for BCG coverage. While BCG coverage increased by 5.85% (*p* = 0.010), significant declines were observed in PHC utilisation (10.53%; *p* = 0.001), non-referred OPD visits (12.05%; *p* < 0.001), and ART commencement (9.53%; *p* = 0.017) rates. Given the importance of PHC in addressing a new pandemic, along with the existing HIV and TB epidemics – as well as the entire quadruple burden of disease – in South Africa, the finding that the PHC utilisation rate statistically significantly decreased in the Free State post-COVID-19 commencement is particularly concerning.

**Conclusions:**

The lessons learned from this retrospective review attest to a measure of resilience in EPHS delivery in the Free State in as far as a significant hike in BCG vaccination over the study period, 2019–2020/21 was observed. As evidenced by a decline in PHC service utilisation and the decreased numbers of new patients commencing ART, we also learned that EPHS delivery in the province was fragile.

## Background

The COVID-19 virus infected more than 600 million and killed more than 6.5 million people [1]. With the end of the pandemic in sight [2–4], it is prudent to assess its effects on health care systems to identify challenges so that a balance between managing emergencies and sustaining essential public health services (EPHSs) can be maintained in the future [5–11].

After declaring the COVID-19 outbreak a global pandemic on 11 March 2020, on 1 June 2020 the World Health Organization (WHO) provided operational guidance to avert indirect morbidity and mortality and prevent acute exacerbations of chronic conditions when services are disrupted. Although the WHO [11] emphasised certain high-priority health service areas – such as essential prevention and treatment services for communicable diseases, including immunisations – it stressed that countries should identify context-relevant EPHSs to be prioritised for continuation during the acute phase of the COVID-19 pandemic.

South Africa embarked on a rapid COVID-19 response, characterised by the temporary suspension of research, diversion of key HIV and TB control resources, and regulation of patient access to healthcare facilities [12]. Early in the COVID-19 pandemic, an assessment of its impact on access to public-sector health care in South Africa indicated a sharp reduction in primary health care (PHC) utilisation [13]. Based on analysis of routine healthcare utilisation data from the District Health Information System (DHIS), this study reported that the pandemic had a considerable influence on healthcare utilisation during April and May 2020 when strict lockdown regulations were in effect. There was a dramatic impact on utilisation of PHC, as well as HIV testing.

Based on data routinely collected via the DHIS in 2019 and 2020, Pillay et al. [14] analysed the impact of the COVID-19 pandemic in South Africa and reported that the severely restrictive lockdowns to reduce transmission and limit the number of patients requiring hospitalisation had mixed consequences for routine health services [14]. While a wide variation of measurable effects on EPHSs were observed, this varied by type of service, province and district.

The full extent of the impact of the COVID-19 on the EPHS spectrum in South Africa is not yet clear. This study aimed to address the need for evidence on the impact of COVID-19 on EPHSs in the Free State province. In order to learn lessons to mitigate the impact of future pandemics on EPHSs, the objectives were to establish the effects of COVID-19 on EPHSs related to 1) PHC and 2) outpatient department (OPD) utilisation, 3) the ART commencement rate, 4) the drug-susceptible tuberculosis (DS-TB) confirmed rate, 5) the DS-TB treatment commencement rate, and 6) BCG coverage during January 2019 to March 2021. The study thus included six indicators on EPHS use and outcomes spanning access to health care services, burden of communicable disease and child health at birth. The PHC utilisation rate was selected as it provided an indication of routine usage of PHC services. Non-referred OPD utilisation was selected as this provided a measure of patients not following the health care service referral policy during the pandemic. ART and TB services were selected as these constitute the largest burden of communicable disease in the province. The DS-TB confirmed and treatment commencement rates relate to the overall global agenda to improve TB diagnosis, care, and prevention [15, 16]. The ART commencement rate relates to one component (care or treatment) of the global agenda to improve HIV diagnosis, care, and treatment [17]. BCG coverage was selected as a significant indicator of hospital services. It is administered at birth or up to one year old in case of stockouts. In contrast, other vaccines are typically administered during infancy at public sector PHC facilities. The selection of BCG coverage also aimed to capture the public–private collaboration that gained prominence during the COVID-19 pandemic. It served as a metric to evaluate the efficacy of private hospitals reporting BCG at birth (a hospital indicator) via the Web-based District Health Information System (Web-DHIS). Considering that other child vaccinations are primarily provided during infancy at PHC facilities free of charge, utilising BCG coverage helped in assessing the impact of public–private collaboration.

## Methods

### Setting

One of the country’s nine provinces, the Free State is located in inland South Africa. The province had a population of 2,928,903 million in 2020 [18] of whom about 80% depend on the public sector for health services [19]. The province is confronted with serious public health systems challenges, including fragmentation of service delivery [20, 21]. Most citizens are historically and socioeconomically disadvantaged due to apartheid spatial and homeland planning, inequality in public funding allocation, social exclusion and segregated access to public sector amenities [20]. In 2016–2021, the province recorded low life expectancy at birth for males (53.0 years in the Free State vs 64.9 years in the best-performing province, the Western Cape) and females (61.4 years in the Free State vs 70.3 years in the Western Cape) [22].

### Routine data sources and data flow into Web-DHIS

**Fig. 1:**
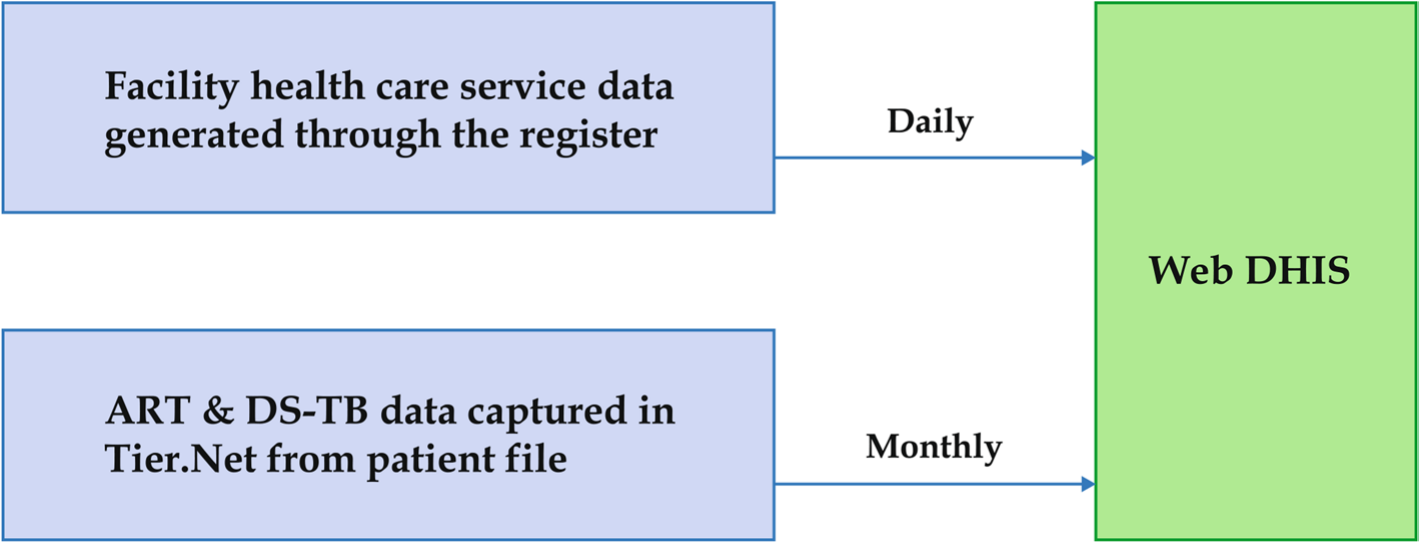
Routine data flow into Web-DHIS

Monthly data from the Web-DHIS for the period of January 2019 to March 2021 (27 months) was used. The data is reported from 220 PHC facilities and 32 hospitals located in the five health districts in the province. A range of performance indicators including BCG coverage (but excluding OPD and PHC utilisation information) is captured daily in Web-DHIS from the registers at all public health facilities in the province. The ART and DS-TB data are captured into Tier.net [23] daily and at the end of each month a report is generated from Tier.net, whereafter the data is captured into Web-DHIS [24]. All the other healthcare services data are captured daily into Web-DHIS from the registers (Fig. 1).

Regarding the quality of the data, it should be noted that data capturing and validation follow the guidelines set by the national DHIS, adhering to standard operating procedures at the facility, district and province levels. In addition, the province has internal controls such as daily data sign-offs in consultation rooms, to enhance data quality. To ensure data plausibility (i.e. data within allowed values and ranges), the health information management units at the provincial and district levels, conduct bi-weekly, systematic validations of newly captured information and communicate with districts and facilities to verify inaccuracies and discrepancies. In this study, the data primarily originated at the provincial level. Notably, there were no missing data regarding critical aspects like the recording of BCG vaccination in the patient file and the Road-to-Health card of the baby. However, challenges remain in ensuring complete recording of data in the register, which serves as the source document for the DHIS data. Otherwise, recorded information is of reasonably good quality.

### Timeline

The first COVID-19 case was reported in South Africa on 5 March 2020, followed by the first COVID-19-related death on 27 March 2020. The state of emergency also begun on 27 March 2020, and containment measures were implemented at the national level including non-essential business and school closures.

### Design and methods

Routinely collected DHIS data were retrospectively analysed using RStudio. The analysis included all data reported through the Web-DHIS and submitted to the National Department of Health from January 2019 to March 2021. Any other routine data collected outside this period was excluded from the analysis. The impacts of the COVID-19 pandemic on EPHS delivery in the Free State were assessed using a pre-post study design, comparing EPHS performance between January 2019 and February 2020 (14 months pre-COVID-19 commencement) and March 2020 to March 2021 (13 months post-COVID-19 commencement). The final dataset comprised indicators on service use and outcomes for each of the 27 months captured at the provincial level.

Interrupted time series analysis was used to assess the median change in service use and outcomes during the first 13 months of the COVID-19 pandemic. Each of the six indicators (PHC utilisation, non-referred OPD utilisation, ART commencement, DS-TB confirmation, DS-TB treatment commencement, and BCG coverage) was regressed on a variable for the COVID-19 commencement period (where 0 is pre-COVID-19 commencement and 1 is post COVID-19 commencement) using time in months to measure the trends. Generalised estimating equation linear models were used to account for repeated observations over time. Median changes were compared using Wilcoxon rank-sum tests. This is an appropriate non-parametric test for comparing the median differences between two independently sampled populations [25]. Interrupted time series analysis using lagged dependent variable model analysis of co-variance (ANCOVA) type II Sum Squares, including a default bootstrap model, was used to confirm observed correlations between time series in the cross-correlation test. A 5% statistical significance level applied.

## Results

Over the study period, the median values were 1) 1.80 for average yearly number of PHC visits per person, 2) 55.30% for non-referred OPD visits, 3) 69.40% for ART-naïve patients commencing treatment, 4) 18.70% for the DS-TB confirmed rate, 5) 95.10% for the DS-TB treatment commencement rate, and 6) 93.70% for BCG coverage. Median changes pre-post COVID-19 commencement are indicated in Table 1. The *p*-values given are obtained from the Wilcoxon rank*-*sum test that was conducted between the pre and post COVID-19 periods.

**Table 1:** Median change in EPHSs measured

Indicator	Median Pre and post COVID-19	Median pre-COVID-19	Median post-COVID-19	Median % change	*P*-value
PHC utilisation rate^a^	1.80	1.90	1.70	-10.53	0.001
Non-referred OPD visits rate^b^	55.30	57.70	50.75	-12.05	< 0.001
ART commencement rate^c^	69.40	71.90	65.05	-9.53	0.017
DS-TB confirmed rate^d^	18.70	19.10	18.10	-5.24	0.297
DS-TB treatment commencement rate^e^	95.10	97.40	93.70	-3.80	0.145
BCG coverage^f^	93.70	91.50	96.85	5.85	0.010

^a^*Definition*: Average number of PHC visits per year per person in the population; *Numerator*: PHC headcount under 5 years + PHC headcount 5–9 years + PHC headcount 10–19 years + PHC headcount 20 years and older; *Denominator*: Population – Total

^b^*Definition*: New OPD clients not referred as a proportion of OPD new clients – total; *Numerator*: OPD headcount not referred new; *Denominator*: OPD headcount not referred new + OPD headcount referred new

^c^*Definition*: All clients started on ART as a proportion of those who tested positive; *Numerator*: ART child naïve start + ART adult naïve start; *Denominator*: All clients tested positive

^d^*Definition*: All DS-TB confirmed as a proportion of TB investigations done; *Numerator*: All DS-TB confirmed clients; *Denominator*: All TB investigations done

^e^*Definition*: All DS-TB treatment started as a proportion of those with confirmed DS-TB; *Numerator*: TB client 5 years and older start on treatment + TB client under 5 years start on treatment; *Denominator*: DS-TB confirmed 5 years and older + DS-TB confirmed under 5 years

^f^*Definition*: Children under 1 year who received BCG, normally given just after birth, as a proportion of population under 1 year; *Numerator*: BCG dose; *Denominator*: Female 00 year + Male 00 year

### PHC utilisation rate

**Fig. 2:**
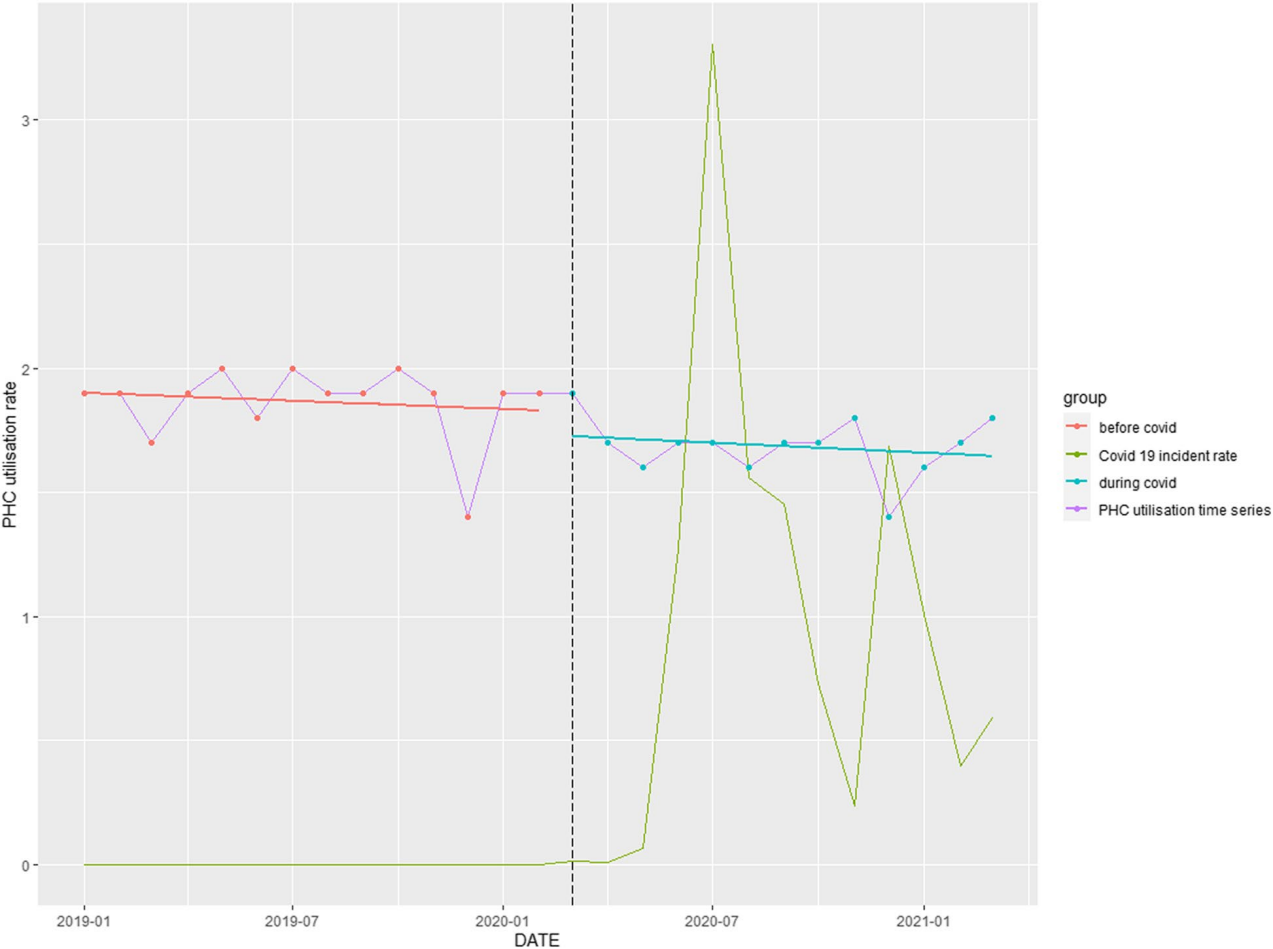
Time series analysis: PHC utilisation

A significant downward trend of 10.53% (*p* = 0.001) over the entire study period was observed (Table 1). Commencement of the COVID-pandemic reinforced the downward trend occurring pre-COVID-19 (Fig. 2).

### Non-referred OPD visit rate

**Fig. 3:**
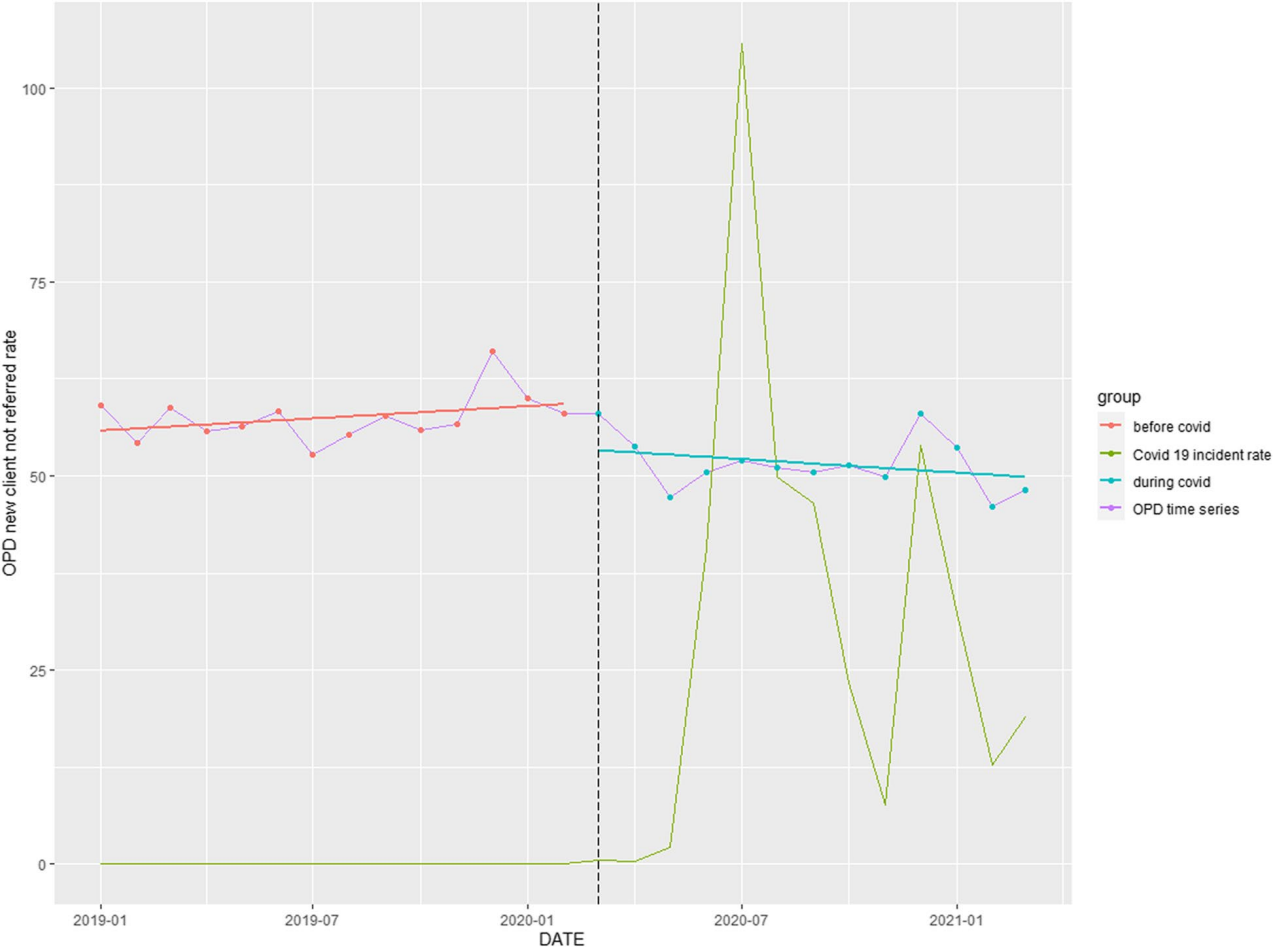
Time series analysis: Non-referred OPD visits

A significant downward trend of 12.05% (*p* < 0.001) over the entire study period was observed (Table 1). Commencement of the COVID-19 pandemic reversed an upward trend occurring pre-COVID-19 (Fig. 3).

### ART commencement rate

**Fig. 4:**
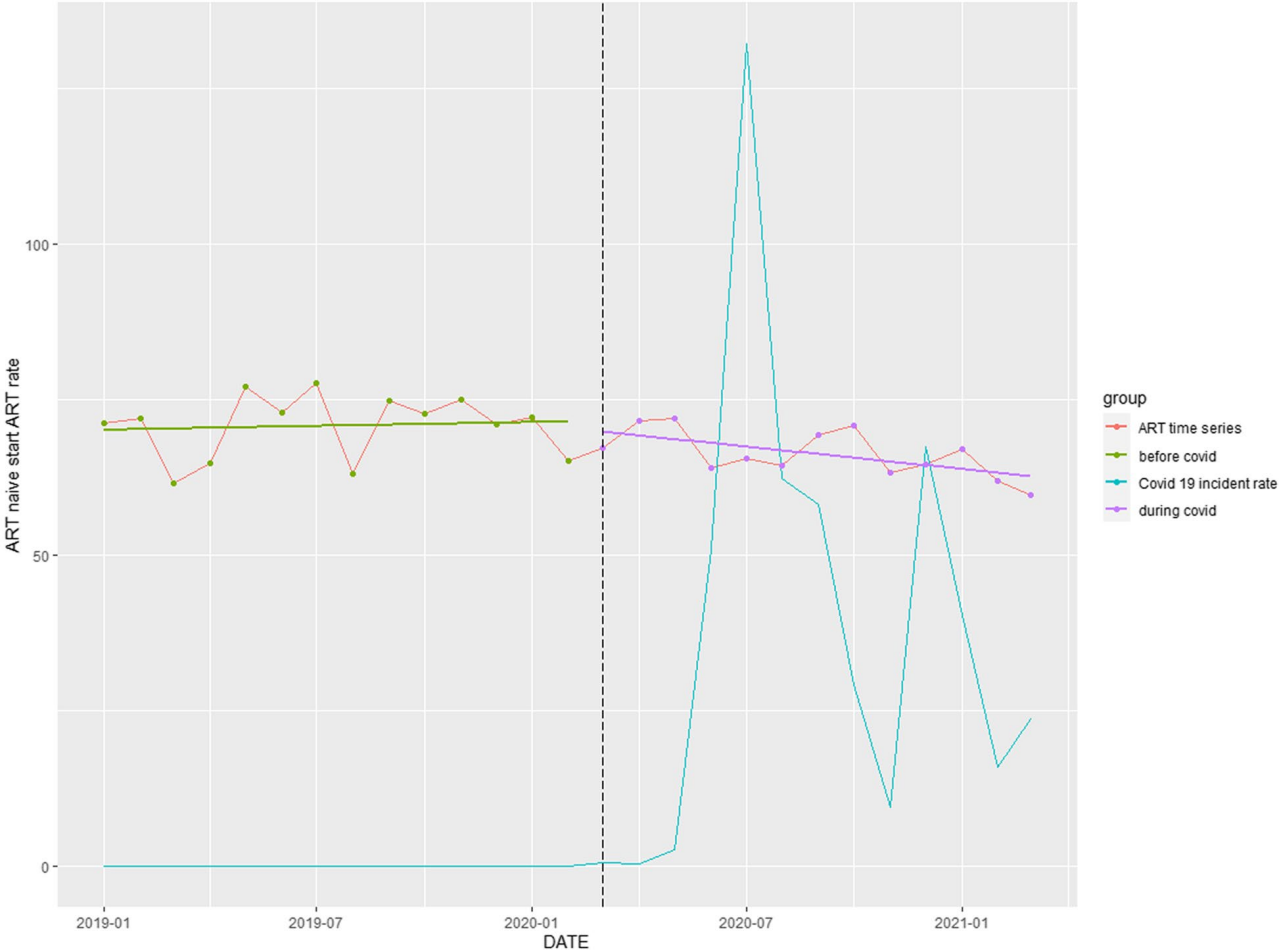
Time series analysis: ART-naïve patients starting treatment

A significant downward trend of 9.53% (*p* = 0.017) over the entire study period was observed (Table 1). Commencement of the COVID-pandemic reversed a slight upward trend occurring pre-COVID-19 (Fig. 4).

### DS-TB confirmed rate

**Fig. 5:**
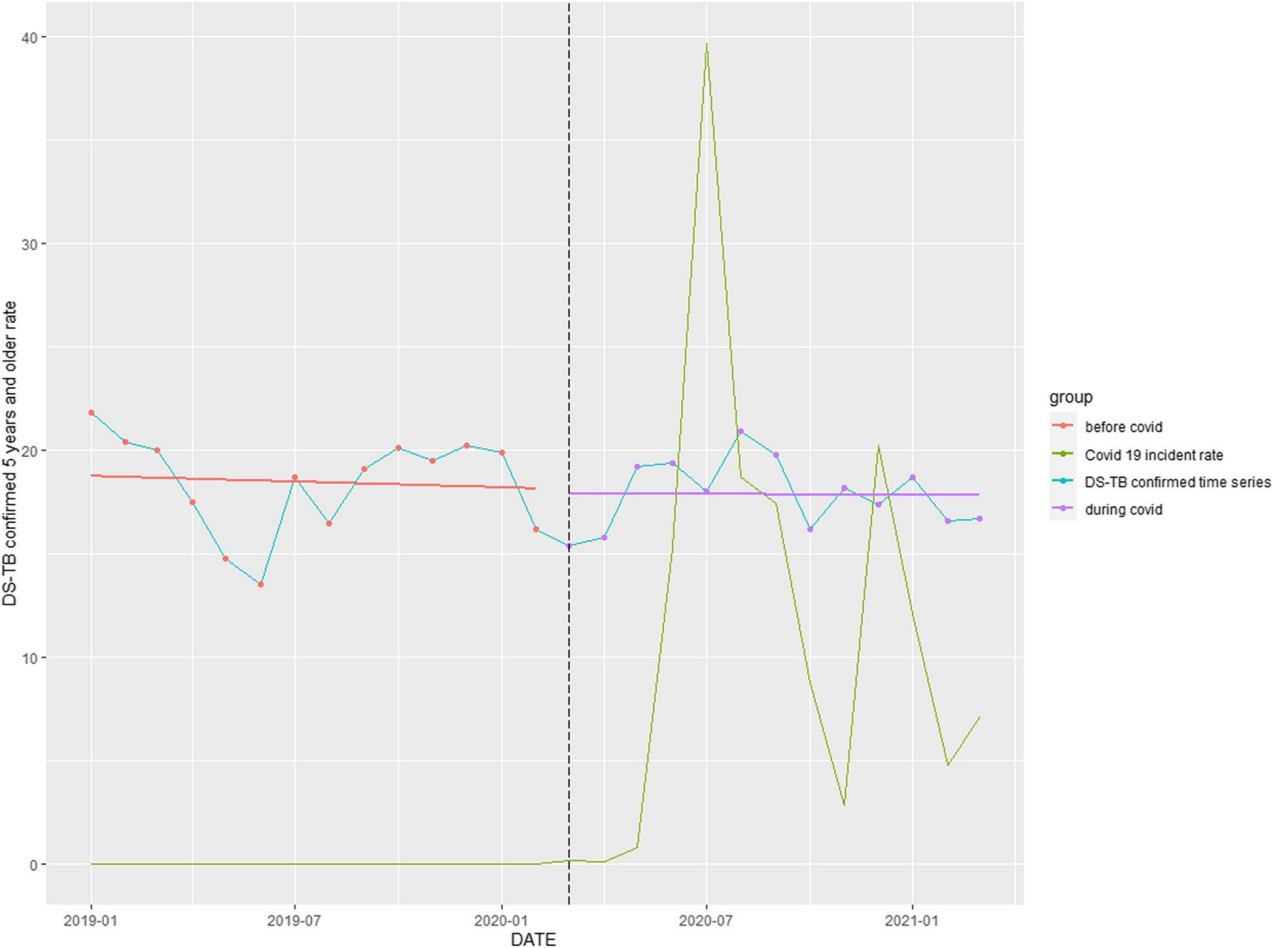
Time series analysis: DS-TB confirmed rate

The DS-TB (≥ 5 years) confirmed rate declined by 5.24% (*p* = 0.297) (Table 1). The COVID-19 pandemic reinforced a slight downward trend occurring before the pandemic (Fig. 5).

### DS-TB treatment commencement rate

**Fig. 6:**
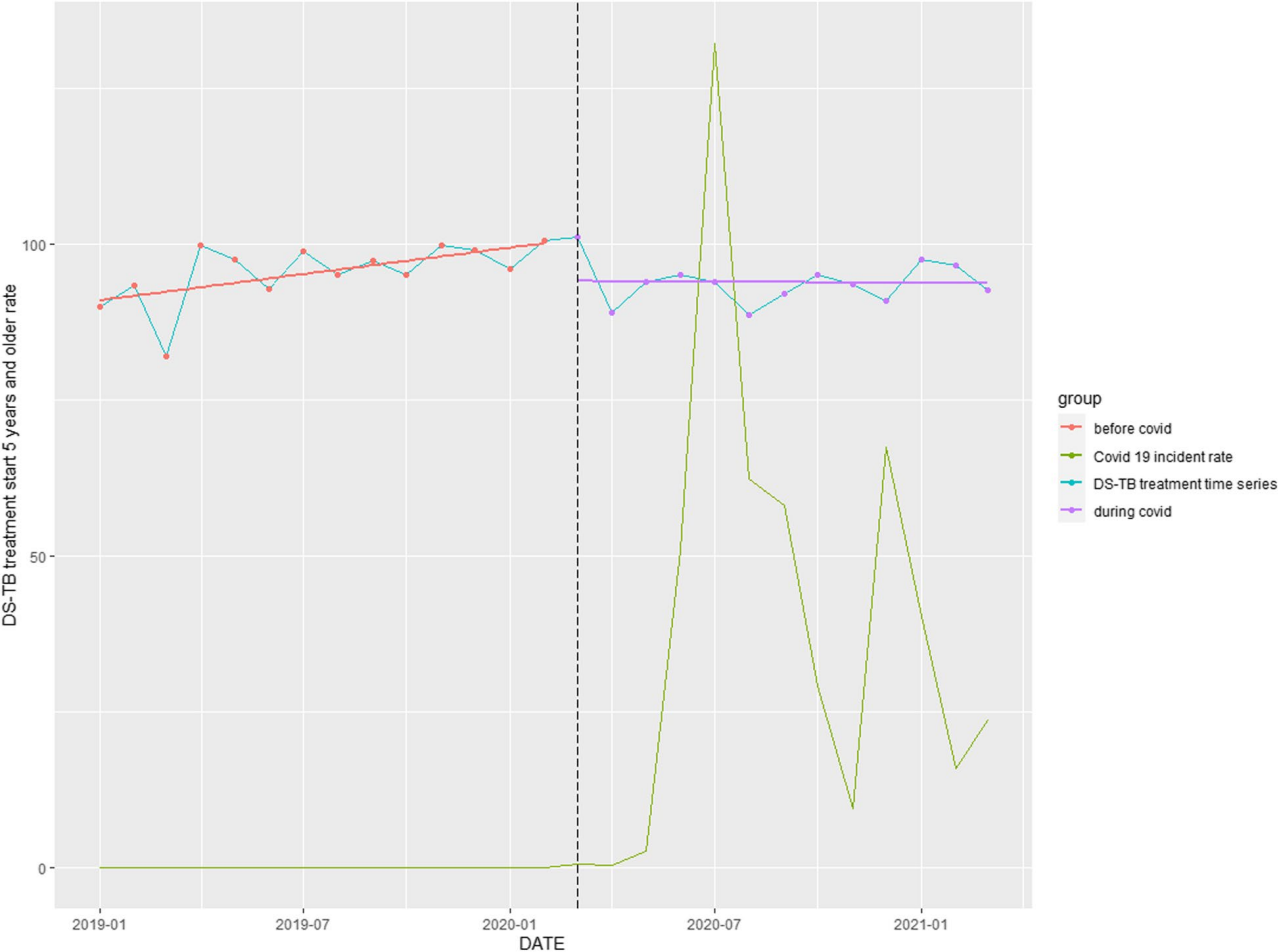
Time series analysis: DS-TB treatment started rate

The DS-TB treatment commencement rate (≥ 5 years) declined by 3.80% (*p* = 0.145) (Table 1). The COVID-19 pandemic reversed an upward trend occurring before the pandemic (Fig. 6).

### BCG coverage

**Fig. 7:**
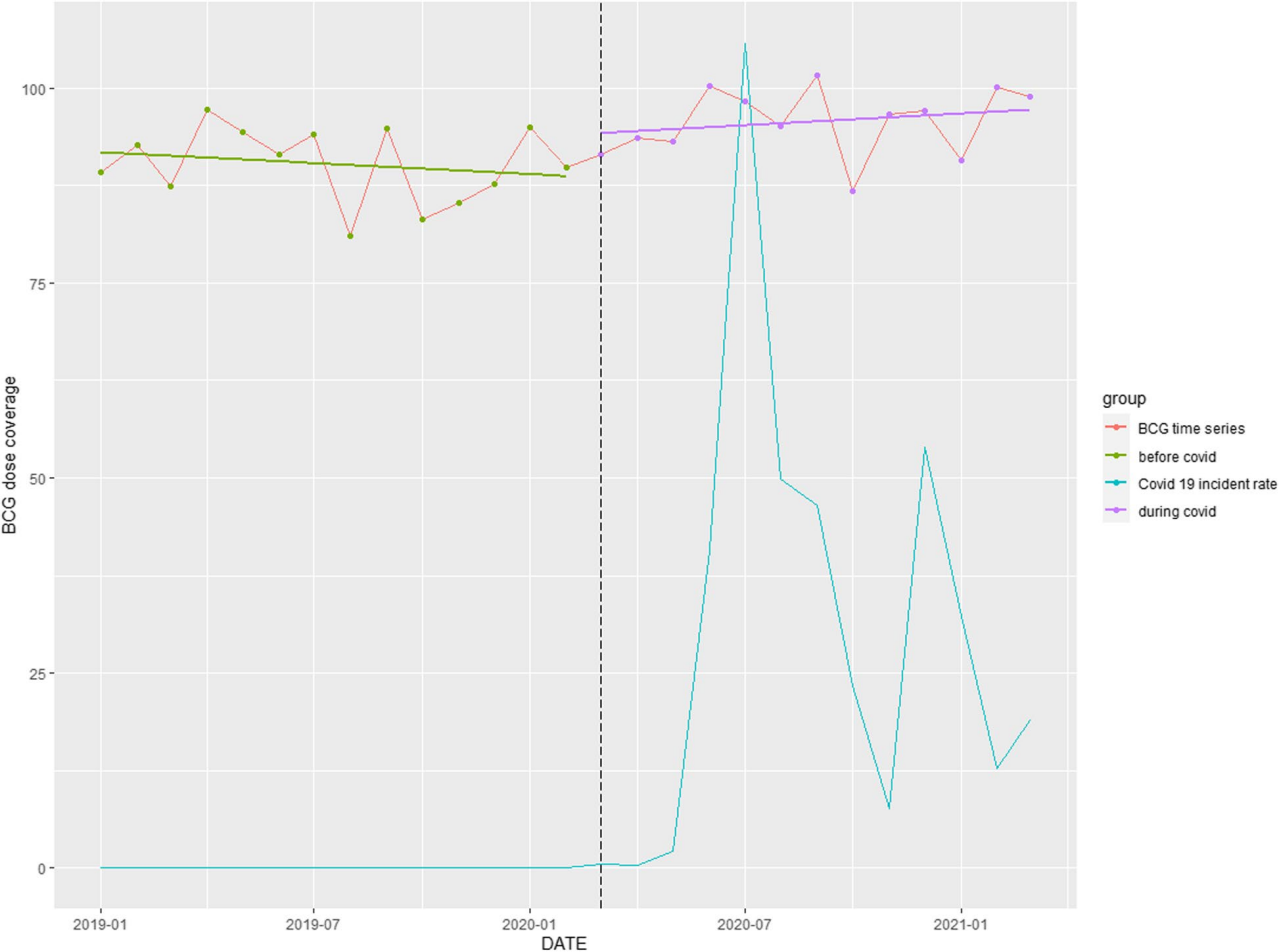
Time series analysis: BCG coverage rate

A significant upward trend of 5.85% (*p* = 0.010) over the entire study period was observed (Table 1). Commencement of the COVID-pandemic coincided with an upward trend occurring pre-COVID-19 (Fig. 7).

## Discussion

This study set out to establish the effects of the COVID-19 pandemic on EPHSs in the Free State province. Decline in EPHSs’ performance during the COVID-19 pandemic in the Free State province raises serious concerns.

Given the importance of PHC in addressing the novel COVID-19 pandemic, along with the existing HIV and TB epidemics – and indeed the entire quadruple burden of disease [26] – in South Africa, the reduction in PHC utilisation post-COVID-19 commencement in the Free State is particularly concerning. Decline in PHC utilisation post-COVID-19 commencement was also reported in Pakistan with a decrease of 12.5% for March 2020 and 33% for April 2020 in the total number of clients who presented for PHC services, as compared to the pre-COVID-19 monthly average [27].

According to Rasanathan and Evans [28], the response to the COVID-19 pandemic showed many missed opportunities for advancing health for all through PHC. The role of PHC was limited in all countries irrespective of income levels. For example, PHC was bypassed for coordinating and conducting specimen collection for testing, community cadres were underused for surveillance, and community engagement and clinical care focused on hospitals. The response demonstrated that health systems in most countries were not sufficiently oriented towards PHC to be mobilised in a crisis.

The COVID-19 pandemic offers important lessons to strengthen health systems through better connection between public health, primary care, and secondary care [29, 30], as well as private care [31–33], to better cope with future waves of this and other pandemics. Local healthcare workers in many countries, came up with creative initiatives such as the introduction or extension of telephone and e-mail use to conduct virtual consulting, and introduced triaging to separate suspected COVID-19 from non-COVID-19 care [29, 34]. However, abandoned or postponed essential and routine care remained a challenge.

Because patients had intentionally avoided health facilities due to fear of contracting these diseases, declines in OPD utilisation were also observed during the Ebola [35] and severe acute respiratory syndrome (SARS) [36] outbreaks. Similarly, declines in OPD utilisation have been observed during the COVID-19 pandemic [37, 38] which portended adverse health outcomes for severely ill patients. Prior to 2020, the lack of an approved referral policy in South Africa resulted in sub-optimal functioning of the referral system with poor linkages and retention in care, a poor continuum of care for patients, and overcrowding of hospitals. In 2020, a national referral policy was released, and defined self-referral as “any person who presents at the hospital/higher level of care for examination, medication or treatment without a referral” [39]. Self-referral to hospitals may be appropriate in the case of major trauma as well as complex conditions but is usually inappropriate in the case of minor acute ailments, chronic conditions, antenatal care or child wellness visits. To counteract and minimise inappropriate self-referrals, hospitals require triaging systems at the outpatient and emergency units. Patients presenting without a referral letter should be assessed, provided with the required treatment, and counselled and referred to the facility closest to their residence. However, medicine may be provided, and no patient should be refused treatment. A bypass fee may be instituted if the patient is an inappropriate self-referral. The decline in non-referred OPD visits in the Free State may thus probably be seen as a positive development, however, this does not apply in the case of appropriate self-referral for trauma and complex conditions. In England, COVID-19 also led to a dramatic fall in the total number of OPD appointments for children and young people, but with a concomitant hike in the proportion of appointments conducted virtually [37].

In high-burden settings, deaths due to HIV, TB and malaria could increase by up to 10%, 20%, and 36%, respectively, compared to a situation without COVID-19 [40]. In respect to HIV, the greatest impact could result from interruption to ART, which could occur during a period of high health system demand. In the Free State, the rate of new ART patients commencing treatment increased before COVID-19, but then fell to an all-time low post-COVID commencement. People living with HIV (PLWH) are one of the groups most vulnerable to COVID-19 [41, 42]. Although research has suggested that PLWH may not be contracting COVID-19 at disproportionate rates (which is hypothesised to be a function of ART), PLWH who are not taking ART or whose disease is not well managed may be at increased risk for contracting COVID-19 due to a compromised immune system and at increased risk for serious symptoms and death [43]. While HIV care delivery was adversely impacted by the COVID-19 pandemic in several countries, it also created opportunities for accelerating effective strategies like multi-month ART [43]. Randomised control trials in southern Africa showed that community-based differentiated service delivery models incorporating three- and six-monthly ART refills and single annual clinical visits were at least non-inferior to standard facility-based care amongst newly stable ART clients aged 25 years or older [44].

From a rapid assessment of national TB programmes across 25 high TB burden countries (including South Africa) in 2022, the Stop TB Partnership highlighted factors contributing to the drop in TB diagnosis, treatment and care [45]. Among others, the disruption of screening, treatment and support activities due to the reallocation of human and physical resources to the COVID-19 response, was projected to have a long-term negative consequence of reversing gains in TB control. Of great concern was that active TB case finding activities had been halted. Also, due to lack of staff and laboratory space, TB diagnosis activities were detrimentally affected. Treatment delay is defined as the time interval between diagnosis and initiation of anti-TB treatment [46]. Delay in the diagnosis and treatment of TB exacerbates the disease and clinical outcomes and further enhances transmission of the infection in the community, as well as increasing the severity of the illness and raising the rate of mortality [47]. The decline of the DS-TB treatment commencement rate in the Free State post-COVID-19 commencement similarly threatened the success of TB control in the province.

Equitable health care implies BCG vaccine for all people in high TB incidence countries and selective vaccination for high-risk populations in low TB incidence countries [48]. Contrary to expectations of an estimated 25% reduction in global BCG coverage within the COVID-19 disruption period [49], BCG coverage in the Free State increased statistically significantly. Possible reasons for this apparent anomaly may include the opportunities created to enhance collaboration between the public and private health sectors in recording BCG coverage. Additionally, in response to BCG stockouts before the onset of COVID-19, a 'catch up' initiative was launched, allowing for the recording of BCG vaccination not only at birth but also in maternity wards using a newly developed form.

Pillay et al.’s [50] research in all nine South Africa’s provinces showed that while by 2021 there was recovery in some EPHS indicators, such as the number of children immunised and HIV tests, in terms of other indicators, including PHC utilisation, the 2019 numbers had yet to be reached. Our study in the Free State confirms the national study’s observation of a slow recovery and a continuing negative impact of the COVID-19 pandemic on EPHSs.

### Limitations

The data analysed are purely quantitative, which prevented deeper exploration of the root causes of the trends noted. Only a limited number (six) of EPHSs were included in the current study, other services may have been affected by the pandemic. It is therefore important to consider other EPHSs in future research to inform the system resilience strategies. The interrupted time series design only controlled for pre-COVID-19 trends, but did not take events other than the pandemic, which may have affected these trends (such as change in policies, funding availability and stockouts) into consideration. The data used in this study are from the Web-DHIS, an administrative data source that may contain errors. However, the information unit staff at district and provincial level conduct regular follow-up with health facilities and systematic validation of indicators.

## Conclusions

The lessons learned from this retrospective review attest to a measure of resilience in EPHS delivery in the Free State in as far as BCG vaccination increased over the study period, 2019–2020/21. However, as evidenced by a decline in PHC service utilisation and the numbers of new patients commencing ART, we also learned that EPHS delivery in the province was fragile. Forthcoming research may consider the WHO’s extended list of sample indicators for monitoring of EPHSs during future pandemics to determine which of these apply and need to be investigated and addressed in local contexts.

## Data Availability

Anonymised data is available from PC on reasonable request.

## Abbreviations

ANCOVA: Analysis of co-variance
ART: Antiretroviral treatment
BCG: Bacillus Calmette-Guérin
DHIS: District Health Information System
DS-TB: Drug-susceptible tuberculosis
EPHS: Essential public health service
OPD: Outpatient department
PHC: Primary health care
PLWH: People living with HIV
TB: Tuberculosis
Web-DHIS: Web-based District Health Information System
WHO: World Health Organization
